# Sarcomeric Pattern Formation by Actin Cluster Coalescence

**DOI:** 10.1371/journal.pcbi.1002544

**Published:** 2012-06-07

**Authors:** Benjamin M. Friedrich, Elisabeth Fischer-Friedrich, Nir S. Gov, Samuel A. Safran

**Affiliations:** 1 Department of Materials and Interfaces, Weizmann Institute of Science, Rehovot, Israel; 2 Max Planck Institute for the Physics of Complex Systems, Dresden, Germany; 3 Department of Chemical Physics, Weizmann Institute of Science, Rehovot, Israel; Emory University, United States of America

## Abstract

Contractile function of striated muscle cells depends crucially on the almost crystalline order of actin and myosin filaments in myofibrils, but the physical mechanisms that lead to myofibril assembly remains ill-defined. Passive diffusive sorting of actin filaments into sarcomeric order is kinetically impossible, suggesting a pivotal role of active processes in sarcomeric pattern formation. Using a one-dimensional computational model of an initially unstriated actin bundle, we show that actin filament treadmilling in the presence of processive plus-end crosslinking provides a simple and robust mechanism for the polarity sorting of actin filaments as well as for the correct localization of myosin filaments. We propose that the coalescence of crosslinked actin clusters could be key for sarcomeric pattern formation. In our simulations, sarcomere spacing is set by filament length prompting tight length control already at early stages of pattern formation. The proposed mechanism could be generic and apply both to premyofibrils and nascent myofibrils in developing muscle cells as well as possibly to striated stress-fibers in non-muscle cells.

## Author Summary

Muscle contraction driving voluntary movements and the beating of the heart relies on the contraction of highly regular bundles of actin and myosin filaments, which share a periodic, sarcomeric pattern. We know little about the mechanisms by which these ‘biological crystals’ are assembled and it is a general question how order on a scale of 100 micrometers can emerge from the interactions of micrometer-sized building blocks, such as actin and myosin filaments. In our paper, we consider a computational model for a bundle of actin filaments and discuss physical mechanisms by which periodic order emerges spontaneously. Mutual crosslinking of actin filaments results in the formation and coalescence of growing actin clusters. Active elongation and shrinkage dynamics of actin filaments generates polymerization forces and causes local actin flow that can act like a conveyor belt to sort myosin filaments in place.

## Introduction

**Figure 1:**
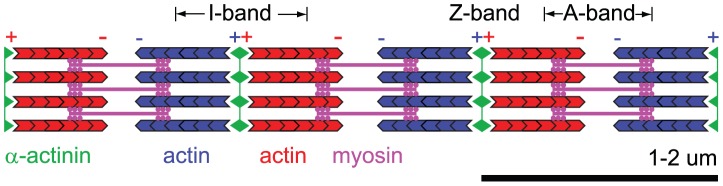
Schematic depiction of sarcomeric organization in myofibrils. Actin filaments (blue and red) are grafted at their plus-ends in an 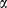-actinin rich crosslinking band, termed the Z-band (green). The repetitive units spanning from one Z-band to the next are referred to as sarcomeres and measure 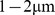 in length. The myosin band (magenta) is traditionally called A-band, while the myosin-free part of the actin band is called I-band. Numerous auxiliary proteins ensure structural integrity and tune elastic properties.

The intriguing striations of muscles were first observed more than a century ago [1]. All skeletal and cardiac muscle cells develop striated acto-myosin bundles of striking regularity termed mature myofibrils, which are characterized by a periodic localization of myosin II filaments alternating with crosslinking regions rich in 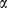-actinin [2]. An analogous, though less regular, arrangement of actin and myosin filaments can be found in adherent, non-muscle cells that express striated stress fibers [3], [4]. Some developing muscle cells contain similar striated stress-fiber like acto-myosin bundles termed premyofibrils and nascent myofibrils [5]–[7] that have been proposed to represent intermediate structures for the formation of mature myofibrils [8]. Figure 1 depicts the periodic structure of mature myofibrils. Periodically spaced crosslinking regions termed Z-bodies or Z-bands delineate 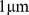-wide sarcomeric regions that comprise actin filaments of organized polarity and crosslinking myosin filaments in the sarcomere midzone. How are these surprisingly regular structures assembled? Numerous proteins involved in myofibrillogenesis have been identified together with their critical role in several muscle diseases [9]. However, the mechanistic basis for sarcomere self-assembly and the establishment of striated order remains elusive. There is evidence that striated fibers are preceded by unstriated fibers, which lack apparent sarcomeric localization of myosin and crosslinkers. Nascent striations first become visible as agglomerations of the actin crosslinker 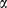-actinin, which then grow and change position to establish a regular, periodic spacing [10]. The formation of these early, unstriated bundles requires the parallel alignment of actin filaments, their mutual crosslinking as well as some means to control bundle thickness. Initial bundle formation depends on actin crosslinking, and possibly Onsager nematic alignment and depletion attractions of entropic origin [11], [12], or kinetic effects due to polar actin flow [13]. The thickness of such actin bundles might be kinetically controlled [14]; additionally, geometric frustration effects due to the chirality of actin filaments have been proposed to set bundle thickness [15]–[17]. Here, we focus on the stage of development in which there is already a pre-formed, unstriated bundle of finite thickness and present a mechanism to explain the subsequent emergence of initial sarcomeric order within this unstriated bundle. In muscle cells, subsequent myofibrillar maturation processes, not modeled here, and fine-tuning of actin filament length, *e.g.* by nebulin [18], [19], drive the transition to final crystalline order.

So far, a number of sarcomeric scaffolding proteins such as titin, N-RAP, and WASP have been identified [18]–[23] and it is highly probable that these scaffolding proteins help to enhance and maintain striated order. However, it is unclear if these scaffolding proteins are able to establish initial striated order in the first place. To do this, these proteins would have to align in a periodic manner on a super-micrometer length-scale by some yet unknown mechanism. Additionally, it is unclear how myosin filaments, which normally walk towards actin plus-ends, become localized near actin minus-ends during myofibril assembly. Here, we ask if physical interactions of actin and myosin filaments, as well as crosslinkers are sufficient to induce initial striated order in filament bundles. Such a mechanism could be generic and could also apply to the formation of striations in acto-myosin stress fibers in non-muscle cells. We show that the combination of treadmilling actin filaments and processive, plus-end tracking crosslinkers suffices to account for the self-organization of striated order and the localization of myosin filaments. Some examples of plus-end tracking crosslinkers such as formins and VASP are known in the biological literature [24], [25]. We emphasize that the plus-end tracking crosslinking of actin filaments in acto-myosin bundles is probably not due tothe action of a single protein, but rather to the concerted assembly by several, interacting structural proteins such as the plus-end capping protein CapZ, the actin crosslinker 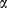-actinin and the giant scaffolding protein titin [26], [27]. Our simple, coarse-grained model replaces this interplay of Z-body proteins by a single “effective” crosslinker that processively grafts actin plus-ends. Note that molecular details may be species-specific: In a recent study by Rui *et al.* [28], the concerted action of the Z-band proteins Zasp, Zipper, kettin, and titin was demonstrated to be pivotal for Z-body formation in Drosophila muscle, while 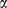-actinin seemed to be dispensable. The strongest evidence for our key assumption of an effective plus-end tracking crosslinker has been provided by recent FRAP-experiments in myofibrils. In these experiments, plus-ends of actin filaments remained localized at the crosslinking band, yet these actin filaments showed polymerization dynamics at their plus-ends. This observation is consistent with the picture of a Z-body acting as a processive, plus-end tracking crosslinker that allows the insertion of new actin monomers while holding the actin filament plus-ends linked with each other. Such a crosslinker could undergo rapid binding and unbinding cycles with actin plus-ends. One study identified a pool of very dynamic actin filaments in mature myofibrils [29]. Physically, a processive plus-end tracking crosslinker results in the condensation of actin filaments into clusters or I-Z-I complexes that consist of two adjacent domains of polarity-sorted actin filaments (I-bands) held together by a crosslinking Z-band, see figure 1. In this paper, we present a minimal model whose analysis shows that actin filament treadmilling and crosslinking can account for the initial establishment of striated order.

### Survey of previous modeling approaches

Several groups have proposed polarity sorting of actin filaments by myosin activity [30], [31]. However, those mechanisms localize myosin filaments close to actin filament plus-ends, which is opposite to the myosin localization observed in striated stress fibers and myofibrils, where myosin resides in the mid region between neighboring crosslinks that attach to the actin plus-ends, see figure 1. In simulations of a generic bundle of polar filaments crosslinked by populations of both plus- and minus-end directed motors, Zemel *et al.* demonstrated sarcomeric ordering with correct polarity sorting if applied to actin bundles [32], see also [33]. However, in the context of actin bundles, there is little evidence for an unconventional, minus-end directed myosin [34].

The concept of a plus-end tracking crosslinker as put forward here has been introduced earlier in the framework of a mean field description [35]. Recently, the group of Joanny proposed a description for the establishment of striated order by stress-induced polarity sorting in terms of a one-dimensional, active gel [36]. However, this mechanism relies on a phenomenological coupling term and as such does not provide insight into the microscopic mechanisms that eventually underlie this coupling.

## Model

### A bundle of treadmilling actin filaments

To describe the transition from an unstriated actin bundle to a striated one, we consider in our simulations a single, long bundle that consists of 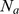 parallel actin filaments aligned with the long axis of the fiber (chosen to be the 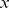-axis). In biological cells, striated fibers have an extension in the transverse direction of only a few hundred nanometers. In our computational model, we therefore ignore the transverse position of the individual actin filaments and assume that each filament can interact with any other provided their projections on the fiber axis overlap. This assumption corresponds to a mean-field treatment of the transverse degrees of freedom.

**Figure 2:**
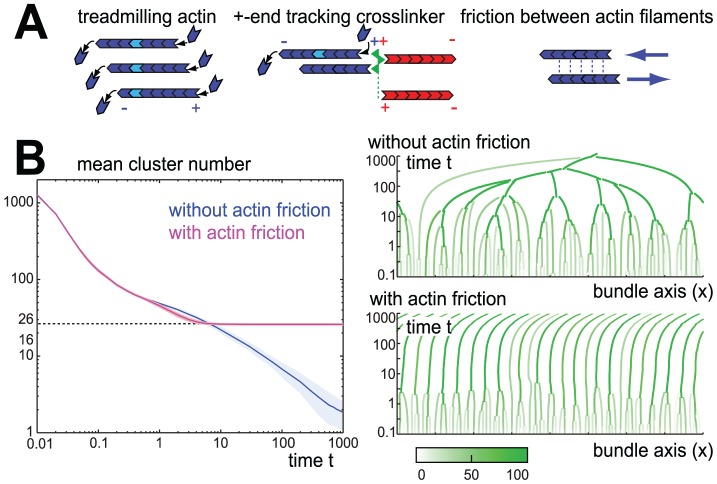
Actin cluster formation and coalescence. **A.** Our computational model of sarcomeric pattern formation considers a one-dimensional bundle of parallel actin filaments, which undergo treadmilling, *i.e.* filaments polymerize at their plus-ends and depolymerize at their minus-ends resulting in a net motion of the plus-end with respect to the individual monomers. Plus-end tracking crosslinkers (green) can permanently attach to the plus-ends of actin filaments (blue and red, indicating filament polarity), while still allowing for polymerization at filament plus-ends. **B.** Plus-end tracking crosslinking results in the formation and coalescence of actin clusters as reflected by a reduction in the number of actin clusters (single actin filaments are counted as one cluster). If there is no friction between sliding filaments (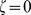), all actin clusters eventually coalesce into a small number of very large clusters (blue, mean
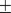
s.e., 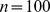). Time is measured in units of actin length divided by treadmilling speed, 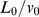. In the presence of inter-filament friction (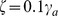), however, actin clusters above a critical size effectively repel each other, resulting in a kinetically stabilized configuration with a finite number of actin clusters (magenta). To the right, example kymographs of actin cluster coalescence are shown for the cases without friction and with friction, respectively. A small imbalance in the number of filaments treadmilling either to the right or to the left within the final striated bundle causes a slow motion of the entire bundle as a whole as is reflected by the tilted cluster trajectories. Using static instead of periodic boundary conditions impedes this motion, see SI text S1. The color scheme encodes filament number in actin clusters as shown in the color bar.

**Figure 3:**
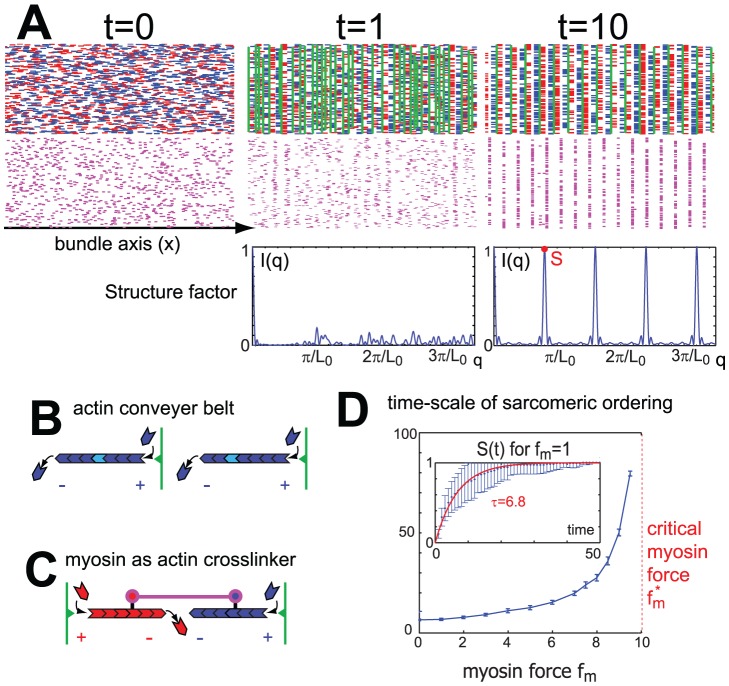
Sarcomeric ordering in the presence of myosin. **A.** Simulation snap-shots showing the emergence of sarcomeric order in an acto-myosin bundle ( single actin filaments: blue and red, myosin filaments: magenta, plus-end crosslinker connecting actin filament plus ends belonging to one cluster : green). Actin filaments can interact if their projections on the bundle axis overlap. Additionally, bipolar myosin filaments (magenta) dynamically attach to actin filaments in a polarity-specific manner, thus acting as a second set of active crosslinkers. Different vertical positions of the filaments are indicated solely for visualization purposes. Initially, filament positions are random (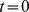). After a transient period during which clusters of crosslinked actin filaments form and coalesce (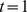), a stable configuration is established characterized by a periodic pattern of actin clusters interspersed by bands of aligned myosin (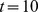). To characterize sarcomeric order in these simulated bundles, we compute the structure factor 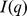 as defined in the main text (blue curves in lower panel, simulation time 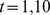, respectively). The height of the principal Bragg peak (red circle) defines the sarcomeric order parameter 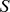. The active myosin force that tends to oppose sarcomeric ordering was chosen as 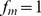, measured in units of 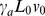. An animated version of this simulation can be found as Video S1 available online as Supplementary Information. **B.** Illustration of the ‘actin conveyer belt’ mechanism: Actin filaments that are grafted at their plus-end by a processive crosslinker have to polymerize against the crosslinker (that acts as an obstacle) and are pushed backwards in a form of local retrograde flow. Myosin filaments interacting with these treadmilling actin filaments are transported away from the cluster center provided that the actin treadmilling speed exceeds the active myosin walking speed. **C.** Myosin filaments that are attached to actin filaments from two neighboring clusters serve as an active crosslinker and mediate repulsive forces between the two clusters due to the difference in the actin polymerization forces and the myosin active forces, see also SI text S1. **D.** Myosin active force slows-down sarcomeric ordering: The inset shows the time-course of the sarcomeric order parameter 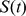 (blue,mean
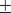
s.e.,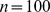) for 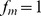, together with a fit of simulation results to an exponential saturation curve 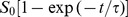 (red) that allows us to extract a time-scale 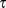 of sarcomeric ordering. The main plot shows this time-scale 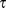 as a function of myosin force 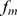; 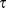 diverges as 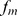 approaches a critical value 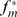. For myosin forces that are larger the critical value 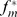, sarcomeric order is not established. Instead, myosin forces facilitate the coalescence of crosslinked actin clusters into a small number of very large clusters (not shown), similar to the case shown in figure 2B without friction.

**Figure 4:**
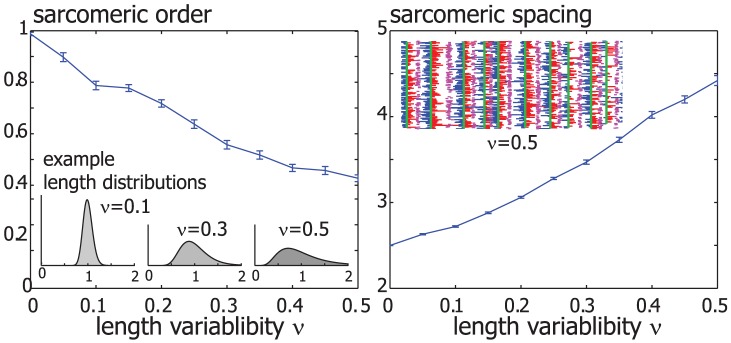
Sarcomeric order despite actin filament length variability. In a modified version of the simulations shown in figure 3, the lengths of individual actin filament were chosen from a unimodular length distribution, see main text. Example length distributions are shown for three values of the length variability parameter 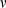. Sarcomeric order also evolved in simulated acto-myosin bundles with a distribution of filament lengths, but with a reduced sarcomeric order parameter and increased sarcomere spacing at steady-state (mean
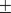
s.e.).

For simplicity, filaments are assumed to be rigid and incompressible with respective lengths 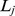, 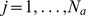. For figures 2, 3, 4, filament lengths are monodisperse with 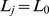 for all 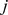; whereas for figure 4 filament length are chosen from a log-normal distribution that satisfies 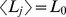 and 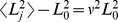, see also the Supporting Information (SI).

Actin filaments are structurally polar and filaments ends are referred to as either the plus-end or the minus end, see figure 2A. We distinguish actin filaments with plus-ends that face either the positive 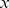-direction (orientation 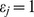, blue in figures), or the negative 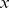-direction (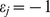, red in figures). Actin filament polymerization is a non-equilibrium process and polymerization and depolymerization rates differ for the plus- and minus-ends, respectively. In a deterministic description of filament polymerization dynamics at steady state, we assume that the individual actin filaments possess a net polymerization speed 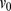 at their plus-ends whose absolute magnitude is equal to the net depolymerization speed at their minus ends. (The corresponding polymerization rate is thus 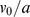, where 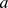 denotes actin monomer length.) The broken symmetry of the polymerization dynamics results in a velocity difference 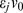 between the current plus-end position 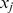 of the 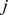-th filament (with a lab-frame velocity 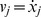) and its individual monomers (velocity 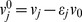). This phenomenon is commonly referred to as filament treadmilling [2], see figure 2A. For an actin filament that is subject only to a friction force 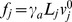 for motion relative to the cytosol, the plus-end advances with velocity 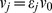, while the monomers are at rest, 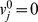, and the friction force 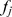 is zero due to force balance. Here, 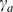 is an effective friction coefficient that accounts for rapid binding and unbinding interactions with the surrounding actin gel, and, possibly, integrin-mediated interactions with a substrate. This situation changes, if rigid crosslinks between actin filaments constrain their motion.

### Processive actin crosslinking

In addition to treadmilling actin filaments, the second key ingredient of our model is a processive, plus-end tracking actin crosslinker that effectively describes the concerted action of several Z-body proteins, see figure 2A. In our simulations, actin filaments become irreversibly crosslinked with a rate 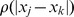, if their respective plus-end positions 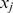 and 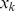 are close. The precise functional form of 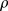 affects results only slightly and we chose 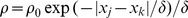 with 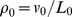 and 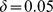 (measured in units of 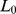). A case of reversible plus-end crosslinking for which actin filaments can spontaneously dissociate again is considered in the SI text S1. Subsequent crosslinking results in the formation of ‘actin filament clusters’ that consist of many actin filaments whose respective plus-ends are aligned and which are permanently crosslinked by effective plus-end tracking crosslinkers. Such an actin cluster will move as a whole subject to the sum of forces acting on its constituent actin filaments. These crosslinked actin clusters can grow by fusion. If two actin filaments belonging to two small clusters establish a new crosslink, the new 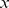-coordinate of the merged cluster is taken as the weighted average of the respective 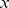-coordinates of the two clusters. In real nascent striated fibers, the longitudinal alignment of plus-ends of crosslinked filaments supposedly involves a dynamic reorganization of the crosslinking Z-band on a time-scale of several minutes [27], which is not included in our minimal model.

Importantly, the proposed plus-end tracking crosslinkers are assumed to be processive, *i.e.* they always remain locally attached to the filament plus-ends, even in the presence of actin treadmilling of the crosslinked filaments, see figure 2A. As a consequence, the center of an actin cluster is subject to polymerization forces of its constituent actin filaments and moves with a velocity 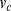 that is determined by a local force-balance of cytosolic friction forces. This force balance is spelled out below in the paragraph ‘Active motion of a single actin cluster’.

For figure 2 only, a generic friction force 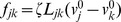 for the relative sliding of two actin filaments is introduced, which is proportional to the mutual length overlap 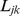 of the two filaments. Here, 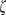 denotes a friction coefficient.

Finally, the motion of actin clusters is determined in each time-step in a self-consistent manner by a balance of forces. We employ periodic boundary conditions with a system size 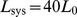; a case of static boundary conditions is discussed in the SI text S1. Total filament numbers were 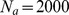 for actin filaments and 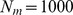 for myosin filaments (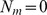 for figure 2).

### Myosin as dynamic actin crosslinker

In the premyofibrils of developing muscle cells as well as in stress fibers of non-muscle cells, the molecular motor myosin II polymerizes into bipolar filaments of a few hundred nanometers length that have numerous myosin heads at either end [37]. Individual myosin heads change conformations via ATP-dependent cycles, while synchronously attaching to (and pushing on) actin filaments. Despite the low duty ratio of individual myosin heads, the large number of these heads ensures a processive and significant myosin-actin interaction. In our simulations, we employ a coarse-grained description of bipolar myosin filaments of length 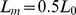, in which the individual myosin heads at the two ends of a myosin filament are described as a pair of ‘actin binding sites’, see figure 3D. Each of these two actin binding sites can bind one actin filament in a polarity-specific way. Attachment and detachment to actin filaments are described as simple Poisson processes with constant rates 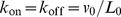. Once a myosin filament is attached to an actin filament, we assume a linear force-velocity relation for myosin walking past the actin filament, see also SI text S1 for details. Myosin walking speed is directly related to an active myosin force 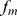 (that also equals the myosin stall force). While myosin filaments tend to walk towards actin filament plus-ends, a strong backward force acting on the actin filament can push both the actin and myosin filaments in the opposite direction. In our simulations, actin treadmilling and associated polymerization forces indeed cause such a motion of myosin filaments towards actin filament minus-ends.

### Active motion of single actin clusters

For sake of illustration, consider an isolated actin cluster that comprises a total number 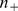 of filaments of positive orientation that treadmill towards the 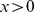-direction (blue in figures) as well as a number 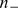 of filaments of negative orientation (treadmilling towards the 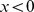-direction, red in figures). In our deterministic description of filament treadmilling, the monomers of the 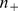 filaments with positive orientation all move with the same velocity 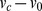, whereas those of the 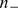 filaments of negative orientation all move with velocity 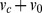. Here 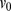 is treadmilling speed and 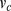 the (yet unknown) velocity of the crosslinking Z-band. The two sets of filaments exert respective friction forces on the cytosol, 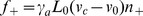 and 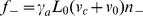, where 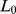 is actin filament length and 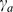 a cytosolic friction coefficient per actin filament unit length, see above. By Newton's third law, the counter forces of these cytosolic friction forces act on the Z-band and amount in this case exactly to the polymerization forces of the treadmilling actin filaments. Local force balance at the Z-band, 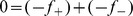, determines the velocity of this single cluster as 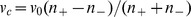.

### The structure factor quantifies sarcomeric order

The structure factor is a standard measure used in condensed matter physics to quantify the regularity of periodic order [38]; it is defined as the squared amplitude of the Fourier transformed density-density correlation function. We can adopt the structure factor to quantify sarcomeric order in our simulations: We characterize the crosslinked clusters by their respective plus-ends positions 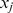 and total filament number 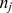. We then define 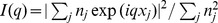. Examples of this structure factor as a function of wave vector 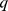 are shown in figure 3A. Periodic order is characterized by a series of very sharp, so-called Bragg peaks. The height 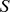 of the principal Bragg peak (red point) defines a sarcomeric order parameter.

### Parameter estimates

Our computational model primarily serves as a proof of physical principle. The emergence of striated order in the framework of this model is a robust process that is not sensitive to the parameter choices. A sensitivity analysis can be found in the SI text S1. Since the parameters in the model represent effective quantities (which, in particular, average out transverse degrees of freedom), numerical estimation of these parameters is difficult. Therefore, our simulation results are presented assuming specific ratios of parameters only, without specifying their absolute values in physical units. Nevertheless, we now present a rough guide to these parameter values.

In unstriated stress fibers, actin filament length range from 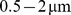, myosin filaments have a length of about 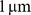 [39]. Thus, the length-scale 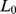, which sets the mean length of actin filaments in our simulations, may be chosen as 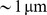. Actin polymerization speeds of up to about 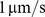 have been observed *in vitro*, while filopodia protrusion driven by actin polymerization can be as fast as 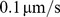, see [40] and references therein. In stereocilia, actin polymerization is highly regulated and polymerization speeds can be as low as 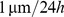 [41]. While in general the polymerization speed of an actin filament is force-dependent with a stall force in the pico Newton range [37], [42], we assume here a constant mean polymerization speed 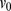. The ratio 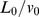 sets the primary time-scale of sarcomeric pattern formation in our simulations, and it is shown below that sarcomeric ordering in established within 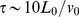 for typical parameter choices. Experimentally, sarcomeric pattern formation evolves on a time-scale of hours [5], which corresponds to an actin polymerization speed 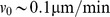 in our simulations. This estimated actin polymerization speed would be lower than that in filopodia, but significantly larger than the speed measured *e.g.* in stereocilia.

Myosin filaments may exert pico Newton forces on actin filament at full activation. Decoration of actin filaments with troponin/tropomyosin reduces myosin walking, which would correspond to lower values for the active myosin force 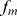 in our simulations. Below, we argue that myosin walking towards actin filaments impedes the correct, sarcomeric polarity sorting, which is established in our model by actin treadmilling. The effective friction for an actin filament moving within a dense bundle is presumably dominated by binding-unbinding interactions with the surrounding actin gel as well as integrin-mediated interactions with the substrate. The corresponding effective friction coefficient 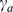 is expected to be orders of magnitude larger than the hydrodynamic friction coefficient for motion in water [43], 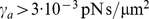. Assuming a friction coefficient for single actin filaments (per unit length) in the range 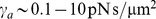, we would find for a filament of length 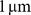 moving at a speed of 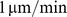 friction forces in the range 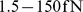, *i.e.* well below both the stall force of actin polymerization and the buckling force of single actin filaments.

We did not incorporate filament diffusion explicitly in our model, as thermal motion will be small in a dense bundle. Note, however, that dynamic myosin forces with short correlation time can induce stochastic, bidirectional motion of filaments.

Several studies pointed out the effect of integrin-mediated anchorage of Z-lines for myofibrillogenesis [44]: Although, initial I-Z-I complexes did form even in the presence of RNAi against integrin, Z-body stability was apparently reduced and bundle integrity was impaired in these experiments [28]. Presumably, integrins play multiple roles starting with the stabilization of I-Z-I-complexes, which corresponds in our model to a reduced rate of dissociation of single filaments from an actin cluster (see also SI text S1). Secondly, anchorage reduces the mobility of I-Z-I complexes, which would correspond to an increased total friction coefficient of actin clusters. As anchored I-Z-I complexes still showed some residual mobility, anchorage must be dynamic and allow for slippage. Thus, dynamic anchorage affects the effective parameters in our model, but does not change its basic, qualitative features. Finally, stable anchorage at the two terminal ends of an acto-myosin bundle specifies its boundary conditions; a simulation case of static boundary conditions is shown in the SI to mimic a bundle whose terminal ends are grafted by focal complexes to a substrate.

## Results

### Plus-end crosslinking facilitates formation and growth of I-Z-I clusters

In our simulations, we consider a minimal, one-dimensional model of a bundle of treadmilling actin filaments. Actin filaments with nearby plus-ends can form a stable crosslink by a complex of molecules (that eventually become the Z bodies) that holds the plus-end of the two actin filaments, but still allows for actin polymerization at the plus-end, see section ‘The computational model’ and figure 2A. Subsequent crosslinking gives rise to the formation of actin clusters that consist of several actin filaments whose respective plus-ends are aligned and which are permanently crosslinked by effective plus-end tracking crosslinkers. Each actin cluster will move as a whole subject to the sum of forces acting on its constituent actin filaments. These crosslinked actin clusters can grow by fusion and eventually self-organize into sarcomeric order, thus representing precursors of the I-Z-I complexes observed during early myofibrillogenesis [45]. To gain basic insight into the process of actin cluster formation and coalescence, we first simulated bundles of treadmilling actin filaments and crosslinks without myosin filaments; the effect of myosin filaments is discussed in the next section. We observe the formation and coalescence of clusters of crosslinked actin filaments, see figure 2B.

In each actin cluster, the constituent actin filaments polymerize at their plus-ends, thereby pushing against the processive crosslinkers of the Z-band. The growing actin filaments themselves move away from the Z-band in a form of ‘local retrograde flow’. The polymerization forces exerted by the polymerizing actin filaments on the Z-band are counter-balanced by friction forces that constrain the motion of the actin filaments. Any imbalance in the number of filaments of the two orientations will result in a net polymerization force and thus net motion of the cluster. The collision of two clusters can result in their mutual coalescence and the formation of a larger cluster. If actin filaments slide past each other without any friction, all filaments would eventually coalesce into a small number of very large clusters, see figure 2B. If we assume, however, a hypothetical, effective friction between moving actin filaments, coalescence of actin clusters above a critical size is dynamically impeded and sarcomeric order results.

The arrest of actin cluster coalescence due to our proposed inter-filament friction can be understood on qualitative grounds as follows: The active motion of a single actin cluster is driven by an imbalance of polymerization forces acting on the Z body that can arise from an imbalance between the respective numbers of the constituent filaments of the two different filament orientations. This net polymerization force is balanced by the total friction force of the actin cluster (and possibly additional forces due to interactions with neighboring clusters). Since this total friction is proportional to the total number of filaments in the actin cluster, whereas the net polymerization force (due to statistical imbalance) roughly scales only as the square root of this number, smaller actin clusters move faster than larger clusters. Furthermore, the mutual friction force between two overlapping actin clusters adds a friction term to the force balance that scales as the product of the respective filament numbers and therefore will eventually stall the approach of actin clusters above a certain size. In the more complex case of an actin bundle, the force balance for all actin clusters has to be considered. Friction between sliding actin filaments may be provided by fast, dynamic crosslinking along the entire lengths of the actin filaments by a second set of crosslinkers. Next, we discuss the possibility that myosin filaments serve as such a dynamic actin crosslinker, which mediates an effective repulsion between neighboring actin clusters.

### Treadmilling actin filaments act as a conveyor belt that moves myosin to the A-band

We now augment the simple actin bundle model by adding bipolar myosin filaments that can dynamically attach to actin filaments in a polarity-specific way, see figure 3D. The relative motion of actin and myosin filaments is governed by a linear force-velocity relation for myosin walking, see section ‘The computational model’. While myosin activity leads to ‘walking’ of the myosin towards the actin plus-ends, the local retrograde flow of treadmilling actin filaments transports the myosin in the opposite direction as in figure 3A. For the case shown, actin treadmilling outpaces active myosin walking towards actin plus-ends, resulting in highly regular sarcomeric patterns with myosin localized near the actin minus-ends. Any actin filament, which is grafted at its plus-end in a Z-band has to polymerize against this obstacle, and is pushed away from the cluster center in a form of ‘local retrograde flow’, see figure 3C. For weak active myosin forces and thus slow active myosin walking, myosin filaments attached to such an actin filament are dragged along with this retrograde flow towards the depolymerizing minus-end of the actin filament. This ‘actin conveyor belt’ not only transports myosin filaments to the future A-band, but also generates an effective repulsion between neighboring I-Z-I clusters mediated by crosslinking actin filaments, which ensures a regular sarcomeric spacing of actin clusters. Stronger active myosin forces drive the myosin towards the actin plus-ends and therefore slow down sarcomeric ordering, see figure 3D. Above a critical force level, active myosin walking dominates actin treadmilling, and a wrong polarity sorting results that localizes myosin at the plus-ends and thus impedes sarcomeric ordering.

### Sarcomeric order despite actin length variability

To account for a distribution of actin filament lengths, we simulated bundles comprising actin filaments of different lengths. For simplicity, we chose a static polydispersity for the actin length given by a unimodular distribution of fixed mean length 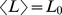 and tunable width 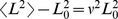. Remarkably, sarcomeric ordering occurred even for considerable length variability 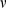, though with a sarcomeric order parameter that decreased monotonically with 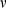, see figure 4. Sarcomeric spacing increased as a function of length variability 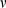, showing that the longest actin filaments set sarcomere spacing. Using an exponential distribution for actin filament length instead of a unimodular distribution resulted in no apparent sarcomeric ordering (not shown). Assuming static filament lengths allows us to study separately the mechanisms that result in actin filament length control and actin turnover, which we now discuss.

### Myosin order despite high actin turnover

**Figure 5:**
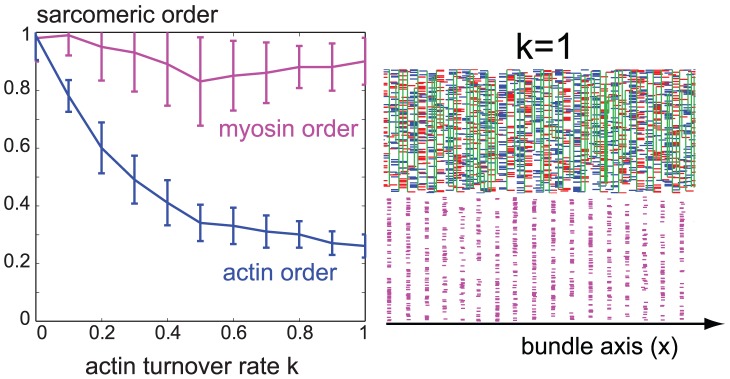
Myosin order despite actin turnover. We devised a minimal model of actin filament turnover, see main text. For simulations as in figure 3, but with actin turnover, the sarcomeric order parameter was found to decrease as a function of actin filament turnover rate (blue curve) as actin turnover impedes the formation of large actin clusters (blue, mean
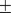
s.e., 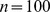). Surprisingly, an analogously defined order parameter for myosin positions attains significant values even for considerable actin turnover rates. A simulation snap-shot at 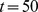 is shown to the right for actin turnover rate 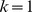 (in units of 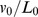).

Actin filament length control and turnover of filaments both depend crucially on the polymerization and depolymerization dynamics of actin filaments. Thus, length control and filament turnover are in principle inseparable. This being said, we nonetheless aimed at isolating the qualitative effect of actin turnover. To this end, we augmented our computational model by including prototypical actin dynamics that differentiates between idealized dynamic regimes of either (i) steady-state treadmilling with constant actin filament length 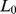, (ii) ‘actin catastrophies’ characterized by fast and complete depolymerization of filaments that occur with rate 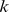, and (iii) rapid *de novo* polymerization of new actin filaments [46]. These simple limits are not intended to realistically depict actin dynamics. Rather they allow us to study the qualitative effects of actin filament turnover, without changing the filament length distribution. As expected, actin filament turnover interferes with the formation of large actin clusters and results in reduced sarcomeric order, see figure 5. Surprisingly, myosin is still sorted into regular A-bands even for considerable actin turnover rates. We conclude that partial polarity sorting of actin filaments is sufficient to sort myosin into A-bands. This may provide an explanation for experimental observations in which myosin ordering was observed to precede the formation of large, periodically spaced I-Z-I complexes.

### A simple model for actin filament length control

**Figure 6:**
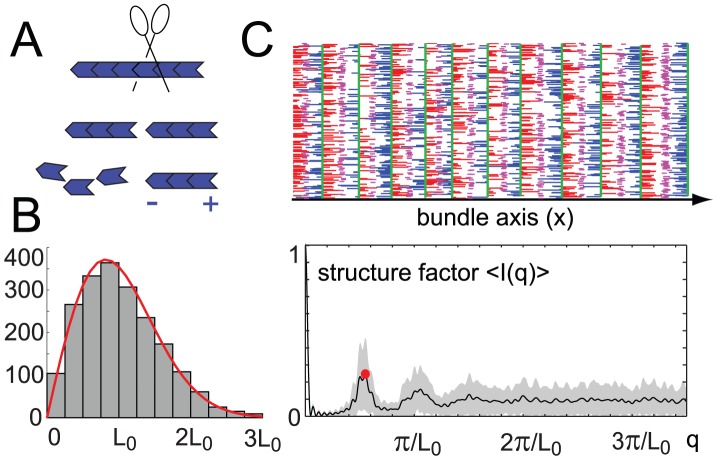
Actin filament length control by severing. **A.** Filament severing provides a simple physical mechanism for actin filament length control, see main text. In an idealized scenario, an actin filament (blue) binds a severing agent (scissors) with a rate 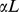 that is proportional to its length 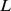 at a random position. The filament is then cut at the binding position, and its minus-end facing fragment is subsequently depolymerized. **B.** Actin filament severing results in a unimodular filament length distribution at steady state, see histrogram (gray) and analytical expression (red, see SI text S1). For the severing rate used, 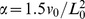, mean filament length 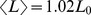, and filament length variability parameter, 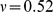. **C.** Simulation of an acto-myosin bundle as in figure 3, but with actin filament severing as described in panel A. Shown is a snap-shot of the simulations at time 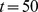 (actin filaments: blue and red; myosin: magenta; end-tracking crosslinker: green), as well as the averaged structure factor (black curve, gray region indicates mean
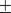
s.e., 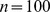).

Our simulations suggest that sarcomere spacing is set by the length of actin filaments at early stages of striated ordering. How is actin filament length controlled within a pool of highly dynamic actin filaments? Capping proteins regulate filament polymerization and depolymerization rates. However, on their own, these proteins do not provide a means to tune the average filament length to a set point since they act locally in a manner that is not sensitive to the total length of a filament. Energetically favorable crosslinking or attraction of actin filaments all along their length can result in a unimodular length distribution as this ensures maximal mutual overlap of filaments [47]. However, to allow for filament sliding and sorting, such crosslinking would have to be highly dynamic. Alternatively, severing agents (such as ADF/cofilin-like UNC-60B [23]) are recruited by actin filaments in a length-dependent manner and can provide a generic feedback mechanism that controls actin filament length [48]–[50]. We consider a simple implementation of actin filament severing assuming that filaments elongate by polymerization at their plus-end with constant polymerization speed 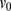, whereas the minus-end is stable. A generic severing agent can bind with constant rate 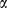 anywhere along the filament and cut it there. Since the minus-end facing fragment of a cut actin filament comprises mainly ADP-bound actin monomers and thus is less stable, we assume that this fragment rapidly depolymerizes after severing, see figure 6A.

This simple severing mechanism results in a unimodular length distribution at steady state, see figure 6B as well as SI text S1. For an intuitive explanation for this length control mechanism, note that longer filaments with more monomers have a higher probability to recruit a severing agent within a certain time interval compared with shorter filaments: In this scenario, filaments act as ‘binding antennas’ for severing agents. Figure 6 shows the emergence of sarcomeric order from an initially unstriated bundle for which actin filaments polymerize and are cut by severing agents.

## Discussion

Here, we proposed a simple, generic, and robust mechanism for striated pattern formation in a crosslinked bundle of aligned actin filaments. This physical mechanism of sarcomeric ordering is based on the formation of small actin clusters by the plus-end crosslinking of single actin filaments and the subsequent coalescence of these smaller actin clusters into larger ones, which are reminiscent of the I-Z-I complexes observed during early myofibrillogenesis [45]. This mechanism represents a way to establish cytoskeletal order on length-scales of tens of microns from micron-size building blocks independent of any external scaffolding. Termination of cluster coalescence and stabilization of sarcomeric units requires a repulsive force between actin clusters. In mature myofibrils, the giant protein titin acts like an elastic spring and could serve this function. However, it is questionable if titin could play its role as a spacer between Z-bodies already at these early stages. While the N-terminal domain of titin is involved in early Z-body formation [28], the M-line epitope of titin associated to its C-terminal domain is established only after a delay [51] and ligand binding may be required to stretch the titin protein so that it spans the sarcomere; thus, at early times, titin may not set the initial sarcomere spacing [20]. Here, we studied polymerization forces from polymerizing actin filaments as a possible mechanism to generate repelling forces between actin clusters. A similar mechanism may apply to stress fibers in adherent, non-muscle cells as well as to stress-fiber like structures in developing muscle cells.

The assembly of mature myofibrils in striated muscle cells has been proposed to be a multi-step process [8] that starts with the formation of unstriated, stress fiber-like acto-myosin bundles near the plasma membrane, followed by the establishment of sarcomeric order within these bundles [10], possibly by actin cluster formation and coalescence as proposed here. These striated bundles represent an important intermediate in the assembly of mature myofibrils and are termed nascent myofibrils. Nascent myofibrils can grow by incorporating free actin and myosin filaments in a mechanism of “self-templating”. Additionally, they can fuse with each other into a single fiber of increased diameter after aligning their respective periodic patterns [5], [52]. Finally, maturation processes and actin length fine-tuning regularizes sarcomeric order resulting in mature myofibrillar “crystals”. This myofibrillogenesis pathway represents a succession of hierarchical ordered states. We speculate that the assembly of striated stress fibers in non-muscle cells may follow a partial sequence of myofibrillar steps. Initial sarcomeric pattern formation in unstriated bundles would be a key step in this pathway and could rely on similar physical mechanisms both in muscle and non-muscle cells.

Experimental visualization of early sarcomeric pattern formation including actin filament length distribution, polymerization dynamics and their associated forces is technically challenging, but may be essential to test theoretical models of sarcomere formation. Little is known about the dynamics of actin filaments at early stages of sarcomeric pattern formation. In mature myofibrils, actin polymerization dynamics has been observed at both the plus- and the minus end [6], [29]. These experiments show that actin filaments are highly dynamic even in these apparently stable striated bundles and that Z-bodies may act as plus-end tracking actin crosslinkers. It should be noted that at these late stages, actin filament treadmilling was not observed; thus, actin treadmilling may be limited to the early stages of striated ordering.

*In vitro* experiments with reconstituted actin stress fibers [53] might serve as an accessible experimental system to study sarcomeric pattern formation and actin polarity sorting. Additionally, filament treadmilling in the presence of crosslinkers is a source of expansive stress and should reduce any contractile prestress in the bundle, or even give rise to an overall expansive stress. This prediction could be tested in future experiments, possibly by laser nano-surgery of unstriated bundles.

Myosin filaments walk towards actin plus-ends. Unless counter-acted by other mechanisms, myosin walking would result in a wrong localization of myosin at nascent Z-bodies and thus impair sarcomeric ordering. In our model, actin treadmilling counter-acts myosin walking and transports myosin towards the future M-band, provided active myosin forces are not too strong. It has been suggested that in some species, the early establishment of sarcomeric patterning involves a non-muscle isoform of myosin II, which is later replaced by muscle-specific myosin II [8]. It is tempting to speculate that muscle myosin allows for maximal force generation, whereas non-myosin filaments play a role as structural elements during the early establishment of striated order, for which, according to our model predictions, strong myosin forces could be obstructive. Alternatively, the decoration of actin filaments with tropomyosin may limit myosin walking during the early stages of sarcomeric pattern formation and thus prevent the active myosin forces from disrupting the treadmilling imposed myosin localization as we suggest. This is consistent with a recent study by Rui *et al.*, which showed that sarcomeric pattern formation was impaired in the presence of RNAi against tropomyosin and troponin [28].

In conclusion, we put forward a model that includes a minimal number of generic mechanisms that results in sarcomeric polarity sorting in *in silicio* acto-myosin bundles. We acknowledge the possibility that the mechanism presented here is only partial and that other mechanisms also contribute to sarcomeric pattern formation that can be tested experimentally. In particular, details of our computational model can differ from the genesis of sarcomeres in developing muscle cells: Actin filament buckling as observed in reconstituted *in vitro* systems [12], [53] may reduce the myosin mediated repulsion force between neighboring actin clusters. Also, adhesive linkage of nascent Z-bodies to an extra-cellular substrate could reduce actin cluster motility [7], [44]. We believe, however, that our theoretical study helps identify key elements of sarcomeric pattern formation. We propose that the length of sarcomere constituents such as actin filaments must be tightly controlled as it is expected to set sarcomere length at early stages of striated ordering. The emergence of sarcomeric order from the active condensation of actin clusters fits into the general framework of cytoskeletal pattern formation by active self-organization, which provides an alternative to external templating mechanisms.

## Supporting Information

Text S1 Supplementary Text S1 provides further details on the computational model used, a sensitivity analysis for the model parameters, a model extension for the case of reversible actin crosslinking, as well as an illustrative mean-field description of actin cluster crosslinking by biopolar myosin filaments.
(PDF)

Video S1Supplementary Video S1 shows the emergence of sarcomeric order in a simulated, one-dimensional acto-myosin bundle: Single, treadmilling actin filaments are shown in blue and red depending on the direction of their plus-end. At their plus end, actin filaments can become permanently crosslinked by a processive crosslinker that tracks actin plus ends while allowing for plus-end actin polymerization. Additionally, bipolar myosin filaments (magenta) dynamically attach to actin filaments in a polarity-specific manner, thus acting as a second set of active crosslinkers. Different vertical positions of the filaments are indicated solely for visualization purposes. Sarcomeric order in these simulated bundles can be quantified by the structure factor 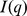 as defined in the main text (blue curves in lower panel). See also figure 3 in the main text.(AVI)
