# Supplementary material for: Sarcomeric Pattern Formation by Actin Cluster Coalescence
Source: PLoS Comput Biol. 2012 Jun 7;8(6):e1002544. doi: 10.1371/journal.pcbi.1002544 (PMC3369942; doi:10.1371/journal.pcbi.1002544)
Supplement: Text S1 — Supplementary Text S1 provides further details on the computational model used, a sensitivity analysis for the model parameters, a model extension for the case of reversible actin crosslinking, as well as an illustrative mean-field description of actin cluster crosslinking by biopolar myosin filaments. (PDF) [file pcbi.1002544.s001.pdf]

# Supporting Information: Sarcomeric pattern formation by actin cluster coalescence

BM Friedrich[1,3,*], E Fischer-Friedrich[2,3], NS Gov[2], SA Safran[1]
**1 Department of Materials and Interfaces, Weizmann Institute of Science, Rehovot, Israel**
**2 Department of Chemical Physics, Weizmann Institute of Science, Rehovot, Israel**
**3 Max Planck Institute for the Physics of Complex Systems, Dresden, Germany**
**∗ E-mail: ben@pks.mpg.de**

## S1 Details of the computational model

### S1.1 Algorithm structure

We employ an Euler scheme with fixed time-step $dt = 0.01L_0/v_0$. (In initial simulations, testing smaller time-steps did not change results.) At the beginning of each simulation, both actin and myosin filaments are assigned random positions $x_j$ and $x_{m,k}$, respectively; additionally, a random orientation $\varepsilon_j = \pm 1$ is assigned to actin filaments. At this stage, no crosslinks between actin filaments exist and all myosin filaments are unbound from the actin filaments.

In each subsequent time-step, any two actin filaments whose projections on the $x$-axis overlap can establish a stable crosslink at their plus-ends with a probability $\rho(|x_j - x_k|)dt = \rho_0 \exp(-|x_j - x_k|/\delta)dt/\delta$ that depends on the distance of the respective plus-end positions, $x_j$ and $x_k$. Subsequent crosslinking results in the formation of 'actin filament clusters' that consist of many actin filaments with aligned plus-ends. If two actin filaments belonging to two small clusters establish a new crosslink, these two clusters then merge into a single cluster. The $x$-coordinate of this new cluster is taken as the weighted average of the respective $x$-coordinates of the two clusters.

In our simulations, an idealized myosin filament with midpoint position $x$ is assumed to have one actin binding site at either end located at $x \pm L_m/2$, where $L_m$ is the length of a myosin filament. Each of these two binding sites can bind to exactly one actin filament in a polarity-specific manner, see figure 3C. (The binding site at $x \pm L_m/2$ binds to an actin filament of orientation $\varepsilon = \pm 1$, respectively.) During a time-step, a free binding site may bind to an actin filament in the range of this binding site with probability $k_{\text{on}}dt$. An occupied myosin binding site may unbind from its actin filament either spontaneously with probability $k_{\text{off}}dt$, or, by forced unbinding, if the depolymerizing minus-end of the actin filament retracts past the binding site.

For the simulations that include actin filament turn-over in figure 5, the number of "actin catastrophies" during a time-step was determined as a Poisson random variable

with mean $kN_a$, where $N_a$ is the total number of actin filaments in the bundle. A corresponding number of actin filaments was randomly selected and removed from the system. Actin filament severing as employed for figure 6 was similarly implemented.

Furthermore, we employ a continuous description of actin polymerization assuming a constant plus-end polymerization speed $v_0$. In a more microscopic description not considered here, this would correspond to a plus-end elongation by four monomers during each time-step (using typical values $L_0 \approx 1\,\mu$m for the length of a two-stranded actin filament and $a = 5.5\,$nm for the size of a monomer [37]).

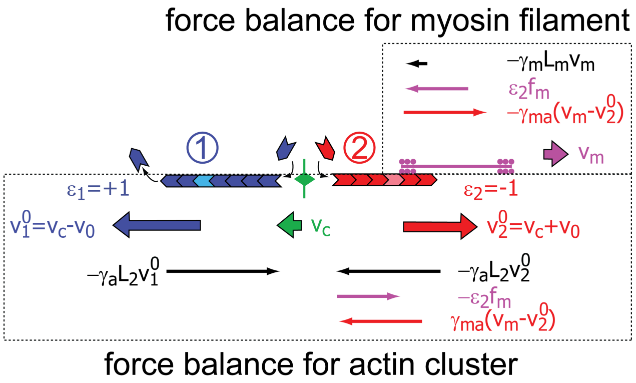


**Figure S1. Local force balances determine velocities.** Motion of actin filaments parallel to the bundle axis is characterized by respective lab-frame velocities $v_j^0$ (of the filament monomers). For actin filaments (red and blue) grafted at their plus-end in a crosslinking band (green), these velocities are offset from the velocity $v_c$ of the crosslinking band by $\varepsilon_j v_0$, where $\varepsilon_j = \pm 1$ denotes filament orientation. Moving actin and myosin filaments are subject to friction with the cytosol; black arrows denote the respective friction forces. The mutual interaction of actin and myosin filaments is modeled by a linear force-velocity relation. As detailed in section S1.3, this relation can be also be represented by an active force $f_m$ of actin-myosin interaction acting on the myosin filament (magenta arrow) as well as a protein friction force $-\gamma_{m,a}(v_m - v_j)$ associated with the interaction (red arrow). Corresponding counter forces act on the respective actin track. Force balance for each crosslinked actin cluster, as well as for each individual myosin filament allows us to self-consistently determine the velocities of all actin clusters and myosin filaments, respectively.

Finally, the individual speeds of actin clusters and myosin filaments inside the bundle are determined in a self-consistent manner by a balance of forces at each actin cluster and myosin filament, respectively. We consider cytosolic friction forces for both actin filaments ($\gamma_a L_j v_j^0$) and myosin filaments ($\gamma_m L_m v_{m,j}$), as well as a linear force-velocity relation for the interaction of bound pairs of actin and myosin filaments, see also SI text. Actin polymerization at the plus-end is taken into account as an offset $\varepsilon_j v_0$ between the velocity

of the actin plus-end $v_j = \dot{x}_j$ and the velocity $v_j^0 = v_j - \varepsilon_j v_0$ by which the individual actin monomers move with respect to the cytosol. The corresponding positions of actin and myosin filaments are updated accordingly in each time-step.

## S1.2 Actin filament length

For figures 2, 3, and 4, we assumed a monodisperse distribution for the length of actin filaments, $L_j = L_0$ for all $j$. An equal polymerization and depolymerization speed $v_0$ at the plus- and the minus-end, respectively, ensures that filament length does not change in time. For figure 4, the length of individual filaments was also taken to be static, but was drawn from a unimodular length distribution $p(L)$ with mean $\langle L\rangle = \int_0^\infty dL\, Lp(L) = L_0$ and variance $\langle L^2\rangle - L_0^2 = \nu^2 L_0^2$. For $p(L)$, we chose a log-normal distribution with scale parameter $\sigma = \sqrt{\ln(1+\nu^2)}$ and location parameter $\mu = -\sigma^2/2$

$$p(L) = \frac{1}{L\sigma\sqrt{2\pi}} \exp\left[-\frac{(\ln(L/L_0) - \mu)^2}{2\sigma^2}\right]. \tag{S1}$$

Finally, for figure 6, the length of individual filaments changes dynamically with time, see section S1.4.

## S1.3 Linear force-velocity relation of actin-myosin interaction

We assume a linear force-velocity relation for the active walking of a myosin filament that is attached to an actin filament. If the actin filament (say of orientation $\varepsilon = +1$) is held fixed with zero velocity ($v_a^0 = 0$), this force-velocity relation reads

$$\tilde{\gamma} v_m = f_m + f_{\text{ext},m}, \tag{S2}$$

where $v_m$ is the velocity of the myosin filament, $f_{\text{ext},m}$ an external force acting on the myosin filament and $f_m$ denotes an active myosin force (that also equals the myosin stall force). The actin filament is subject to an opposite force $-f_m$. The coefficient of proportionality $\tilde{\gamma} = \gamma_m L_m + \gamma_{m,a}$ represent a friction coefficient that combines a contribution stemming from a cytosolic friction force $\gamma_m L_m v_m$ for myosin motion relative to the cytosol, and a contribution that effectively describes protein friction of the actin-myosin interaction, $\gamma_{m,a}(v_m - v_a)$, which we assume is proportional to the relative velocity of the myosin with respect to the actin. We use $\gamma_a = \gamma_m$ and $\gamma_{m,a} = 10\gamma_a L_0$. The above force-velocity relation can thus be rephrased in equivalent form as a force balance

$$\gamma_m L_m v_m + \gamma_{m,a}(v_m - v_a^0) = f_m + f_{\text{ext},m}. \tag{S3}$$

This formulation generalizes in a straightforward manner to the case of a moving actin filament. If $f_{\text{ext},a}$ denotes an external force acting on the actin filament of length $L_a$, we have an analogous force balance for the actin filament

$$\gamma_a L_a v_a^0 - \gamma_{m,a}(v_m - v_a^0) = -f_m + f_{\text{ext},a}. \tag{S4}$$

In the context of our actin bundle simulations, the external force $f_{\text{ext},a}$ is actually zero for free actin filaments. For polymerizing actin filaments grafted in a Z-band, however, $f_{\text{ext},a}$ is non-zero and represents the counter force of the actin polymerization force. Given $f_{\text{ext},a}$ and $f_{\text{ext},m} = 0$, we can self-consistently solve for the myosin and actin velocities, $v_m$ and $v_a$, respectively, see figure S2. In the absence of external forces, the active myosin force $f_m$ causes the myosin filament to move towards the actin plus-end, while the actin filament itself is pushed backward as a result of a counter-acting force $f_m$. A strong, backward-directed external force acting on the actin filament, $f_{\text{ext},a} < -\gamma_a/\gamma_{m,a} f_m$, pushes both the actin filament and the myosin filament backwards, $v_a, v_m < 0$, despite the fact that the myosin filament advances relative to the actin filament, $v_m - v_a > 0$.

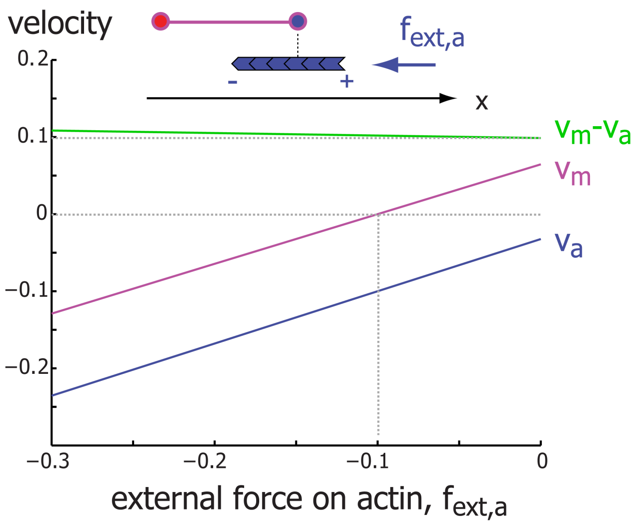


**Figure S2. Force-velocity relation of actin-myosin interaction.** The walking of a myosin filament (magenta) with respect to an actin filament (blue) is modeled by a linear force velocity relation, see section S1.3. A backward directed force $f_{\text{ext},a}$ acting on the actin filament can push both the actin and the myosin filament backward (in the $-x$-direction). Such forces arise as counter forces of actin polymerization forces in our simulations. In this case, the myosin filament continues to advance with respect to the actin filament as indicated by a positive velocity difference $v_m - v_a > 0$.

## S1.4 Actin filament length control by severing

We present a simple model for the length control of polymerizing actin filaments, which is employed in a modified version of our computational model presented in figure 6. This simple model idealizes a more sophisticated model discussed in [48–50]. We consider a pool of $N_a$ filaments, which for simplicity are assumed to elongate at their plus-end with

a constant polymerization speed $v_0$, while their minus-ends are stable (possibly due to minus-end capping). Consequently, the length of a filament grows in time, $\dot{L}_j = v_0$. Additionally, severing agents can bind to a filament with equal probability all along the filament length and cut the filament at the binding position, see figure 6A. Subsequently, the minus-end facing fragment of the cut filament is assumed to depolymerize completely (possibly due to the fact that it consists mainly of ADP-actin), whereas the plus-end facing fragment remains (possibly recruiting a new cap for its minus-end). Let $\alpha\, dL$ denote the rate by which a severing agent binds to a short length segment $dL$ of a filament. Then the total probability that during a short time-interval $dt$ a filament of length $L$ is cut somewhere along its length is $\alpha\, L\, dt$, *i.e.* the overall scission probability is proportional to filament length. A simulation of this mechanism with $N_a = 2000$ actin filament results in unimodular distribution of filament length at steady-state, see figure 6B.

In a mean-field description, the filament length distribution $p(L)$ is found to obey a master equation

$$\frac{\partial}{\partial t} p(L,t) = -v_0 \frac{\partial}{\partial L} p(L,t) - \alpha\, L\, p(L,t) + \alpha \int_L^\infty dl\, p(l,t). \tag{S5}$$

The first term on the right hand side is a convective term that arises from the polymerization speed and describes a flux in probability space due to the elongation of actin filaments by polymerization. The second term is the rate at which filaments of length $L$ are cut into smaller filaments by the severing agent, which decreases the number of filaments of length $L$. The third term finally represents the rate of accrual of stable, plus-end facing fragments of size $L$ from the scission of longer filaments. The probability that a cut will occur at a distance $L$ from the plus-end of a long filament of length $l$ is $\alpha$. Equating the left-hand side of equation (S5) to zero, we can solve for the the steady-state length distribution $p_0(L)$ and find

$$p_0(L) = \alpha L/v_0 \exp[-\alpha L^2/(2v_0)]. \tag{S6}$$

The mean filament length $\langle L \rangle$ is determined by a competition of the cutting rate $\alpha$ and the polymerization speed $v_0$, whereas the normalized length variability $\nu$ is independent of both $\alpha$ and $v_0$

$$\langle L \rangle = \sqrt{\frac{\pi v_0}{2\alpha}}, \quad \langle L^2 \rangle - \langle L \rangle^2 = \nu^2 \langle L \rangle^2, \quad \nu = \sqrt{(4/\pi) - 1} \approx 0.52. \tag{S7}$$

# S2 Model parameters and sensitivity

Table S1 lists reference parameters used for the figures (unless stated otherwise). The parameters marked with an asterisk $(L_0, v_0, \gamma_a)$ set a characteristic length scale $(L_0)$, time-scale $(L_0/v_0)$, and force-scale $(\gamma_a L_0 v_0)$, respectively. All other parameters are defined in

| symbol | meaning | value | range |
| --- | --- | --- | --- |
| $N_a$ | number of actin filaments | 2000 | 250-4000 |
| $N_m$ | number of myosin filaments | 1000 | 250-2000 |
| $L_0$ | mean actin filament length | 1 (*) | n.a. |
| $L_m$ | myosin filament length | 0.5 ($L_0$) | 0.1-1 [a] |
| $L_{\text{sys}}$ | system size | 40 ($L_0$) | 20-80 |
| $v_0$ | actin polymerization speed | 1 (*) | n.a. |
| $\gamma_a$ | cytosolic friction coefficient for actin | 1 (*) | n.a. |
| $\gamma_m$ | cytosolic friction coefficient for myosin | 1 ($\gamma_a$) | 0-10 |
| $\gamma_{ma}$ | friction coefficient for actin-myosin interaction | 10 ($\gamma_a L_0 v_0$) | 2.5-100 |
| $f_m$ | active myosin force | 1 ($\gamma_a L_0 v_0$) | 0-9.5 [b] |
| $k_{\text{on}}$ | actin-myosin binding rate | 1 ($v_0/L_0$) | 0.05-10 |
| $k_{\text{off}}$ | actin-myosin unbinding rate | 1 ($v_0/L_0$) | 0-10 |
| $\delta$ | range of actin crosslinking | 0.05 ($L_0$) | 0.01-0.1 |
| $\rho_0$ | base rate of actin crosslinking | 1 ($v_0/L_0$) | 0.01-1 |

[a] System size was adapted as $L_{\text{sys}} = 16(2L_0 + L_m)$ to be an integer multiple of the expected sarcomere size.

[b] A simulation time of $t = 250$ was chosen to account for an increased time-scale of sarcomeric ordering.

**Table S1.** Reference parameters used in simulations.

a dimensionless manner relative to these scales. We independently varied parameters and determined (non-exclusive) ranges for which robust sarcomeric pattern formation occurred (characterized by a mean sarcomeric order parameter $\langle S \rangle > 0.9$; simulation time $t = 50$).

# S3 Periodic and static boundary conditions

The simulations *in silicio* acto-myosin bundles presented in the main text employ periodic boundary conditions. An example kymograph of actin cluster formation and coalescence for periodic boundary conditions is shown in figure S3A. For periodic boundary conditions, a small drift of the entire bundle may occur at steady state as a result of a small imbalance in the number of actin filaments of the two orientations. In real nascent myofibrils, the boundary conditions for pattern formation are defined by integrin mediated anchorage at the two terminal ends of the myofibril. These focal complexes locally organize actin polarity. To mimick this situation, we ran simulation, where the terminal ends of the bundle are represented by non-moving actin half-clusters of organized polarity, see figure S3B. Without proper boundary conditions, sarcomeric ordering is impaired in our simulations.

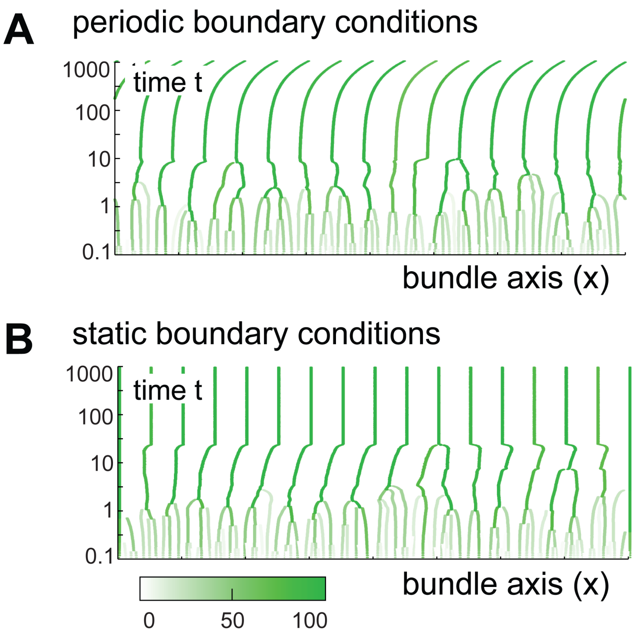


**Figure S3. Changing boundary conditions. A.** Kymograph of actin cluster formation and coalescence in the presence of myosin for the simulation shown in figure 3 of the main text. This simulation employed periodic boundary conditions. **B.** Kymograph of actin cluster formation and coalescence in a simulated acto-myosin bundle as in panel A, but for static boundary conditions. Static boundary conditions are realized by inserting two actin half-clusters at the two bundle ends whose positions are fixed throughout the simulation, $x_1 = 0$ and $x_2 = L_{\mathrm{sys}}$, by imposing suitable constraining forces. Each half-cluster comprises $N = 50$ actin filaments of specified polarity at $t = 0$. This mimics bundles that are grafted by focal adhesions at their terminal ends. The color scheme encodes filament number in actin clusters as shown in the color bar.

# S4 Reversible crosslinking of actin filaments

Prompted by the experimentally observed stability of I-Z-I complexes, we assumed in the main text that actin filaments become irreversible crosslinked at their plus-ends to form actin clusters. We now relax this assumption, allowing for dissociation of single actin filaments from a crosslinked actin cluster with a finite rate $\rho_{\mathrm{off}}$. We find that robust sarcomeric pattern formation persists provided the effective binding rate $\rho/\delta$ exceeds the unbinding rate $\rho_{\mathrm{off}}$, see the phase diagram in Fig. S4.

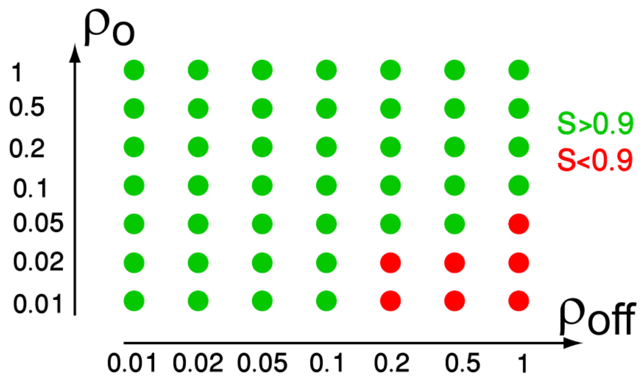


**Figure S4. Sarcomeric ordering for reversible actin crosslinking.** We re-ran simulations with the standard parameter set (and $f_m = 0$), but now allowed for the dissociation of single actin filaments from a crosslinked actin cluster with a rate $\rho_{\text{off}}$ to mimic the reversible crosslinking of actin filaments. Shown is a phase diagram of sarcomeric ordering as a function of the binding rate $\rho_0$ and the unbinding rate $\rho_{\text{off}}$. (To classify order, we used the criterion $S > 0.9$ for the mean structure factor $S$ averaged over $n = 100$ simulations.) The ordering is affected by the reversibility of the crosslinking only when the unbinding rate is large compared to the binding rate.

# S5 Myosins crosslink actin clusters

In our model, bipolar myosin filaments can mechanically link neighboring actin clusters by binding to one actin filament from each cluster, respectively. These linker myosin mediate an effective interaction force between the two clusters: While myosin tends to walk towards actin plus-ends, thus pulling the two clusters closer together as in the sliding filament model of sarcomere contractions, actin treadmilling together with acto-myosin friction mediates an effective repulsion. For a sufficiently high density of actin filaments, the net repulsion force between the two clusters scales with the total number $n$ of linker myosins, $(\gamma_{m,a} v_0 - f_m) n$. Figure S5A shows the number of myosin filaments linking two neighboring actin clusters as a function of the separation distance $\Delta x$ between cluster centers in simulations with variable actin filament length; this dependence is non-monotonic. Intuitively, this can be understood as follows: For small separation distances, only a small number of myosin filaments happen to be enclosed between two clusters. For large separation distances, however, the number of long actin filaments that can possible engage in a myosin-mediated mechanical link is small. In the following, we will make this reasoning more quantitative. The expected total number of myosin filaments fully enclosed by two cluster centers can be approximated by $c_m(\Delta x - L_m)$, where $c_m = N_m / L_{\text{sys}}$ is the density of myosin filaments in the bundle and $\Delta x > L_m$ the separation distance of the two clusters. Out of this total number of myosin filaments between the two clusters, only a

certain fraction will actually bind to two actin filaments at a given time. We can estimate this fraction of linker myosin filaments by formulating a mean-field theory. For this, we consider an idealized scenario of two static actin half-clusters separated by a distance $\Delta x$, each of which comprises a number $N$ of actin filaments whose individual lengths are distributed according to some length distribution, $p(L)$, see figure S5B. We characterize the myosin filaments enclosed between the two cluster centers by four different concentration fields of their midpoint positions, according to whether they are not bound to any actin filament $[c_0(x)]$, bound to an actin filament from either the left or right cluster only $[c_L(x),\ c_R(x)$, respectively], or, if they are true linker myosins that are bound to actin filaments from both the left and the right cluster $[c_2(x)]$. The dynamics of these concentration fields is governed by convection due to the actin conveyor belt with speed $v_m = (\gamma_{ma} v_0 - f_m)/(\gamma_{ma} + \gamma_m)$, as well as by exchange terms $\Delta_*$ due to binding/unbinding kinetics and forced unbinding of myosins that have reached the depolymerizing minus-end of an actin filament

$$\begin{aligned}
\dot{c}_L &= -v_m \nabla c_L - \Delta_{L2} - \Delta_{L0}, \\
\dot{c}_R &= +v_m \nabla c_R - \Delta_{R2} - \Delta_{R0}, \\
\dot{c}_0 &= \qquad\qquad\qquad + \Delta_{L0} + \Delta_{R0}, \\
\dot{c}_2 &= \qquad\quad + \Delta_{L2} + \Delta_{R2},
\end{aligned} \tag{S8}$$

where the exchange terms read

$$\begin{aligned}
\Delta_{L0} &= k_{\text{off}} c_L - k_{\text{on}} \Phi(x_L) N c_0 + v_m \frac{p(x_L)}{\Phi(x_L)} c_L, \\
\Delta_{R0} &= k_{\text{off}} c_R - k_{\text{on}} \Phi(x_R) N c_0 + v_m \frac{p(x_R)}{\Phi(x_R)} c_R, \\
\Delta_{L2} &= -k_{\text{off}} c_2 + k_{\text{on}} \Phi(x_R) N c_L, \\
\Delta_{R2} &= -k_{\text{off}} c_2 + k_{\text{on}} \Phi(x_L) N c_R.
\end{aligned} \tag{S9}$$

Here, $x_L = x - L_m/2$, $x_R = \Delta x - x - L_m/2$, and $\Phi(x) = \int_x^\infty dx'\, p(x')$ is the cumulative distribution function of actin filament lengths that counts how many filaments have sizes greater than $x$. The exchange rate $\Delta_{L0}$ characterizes the exchange between the pool of myosin filaments exclusively attached to an actin filament from the left cluster and the pool of free myosin filaments that are not bound to any actin filament: Spontaneous unbinding occurs occurs at a rate $k_{\text{off}} c_L(x)$, while the rate of binding of free myosin filaments with center position $x$ to an actin filament from the left cluster is proportional to the number $\Phi(x_L)N$ of actin filaments that are long enough to extend to position $x_L$, where $x_L$ is the position of the left binding site of these myosin filaments. The latter rate thus reads $k_{\text{on}} \Phi(x_L) N c_0(x)$. Finally, the third term accounts for forced unbinding of myosin filaments: once a myosin reaches the depolymerizing minus end of its actin track, it 'falls off' the filament to which it was bound. Forced unbinding of myosin filaments occurs

with a rate $v_m[p(x_L)/\Phi(x_L)]c_L$ that is proportional to the local proportion $p(x_L)/\Phi(x_L)$ of actin filaments of length $L = x_L$ among those with a length larger than $x_L$. The other exchange rates are derived similarly. Provided none of the actin filaments extends over the entire cluster distance, myosins are confined to the region between the clusters and a steady state evolves. At steady state, we find

$$c_2(x) \sim \frac{k_{\text{on}}N}{k_{\text{off}}}\, F_+ \, \exp\left[\frac{k_{\text{on}}N}{v_m}\, F_- + \frac{F_-}{F_+}\left(\frac{k_{\text{off}}}{v_m} + G_1\right) - G_2\right] \tag{S10}$$

Here, we used short-hand notation $F_\pm = (1/2)[\Phi(x+L_m/2)\pm\Phi(\Delta x-x-L_m/2)]$ and $G_\pm = (1/2)[p(x+L_m/2)/\Phi(x+L_m/2)\pm p(\Delta x-x-L_m/2)/\Phi(\Delta x-x-L_m/2)]$. Figure S5B shows the analytical solution from eq. (S10), revealing the formation of a myosin band in the midzone between the two actin clusters at steady state. Interestingly, this steady state is characterized by a cyclic flux of myosin filaments, see figure S5B: Myosins bound to a blue actin filament are actively transported to the left until they detach either spontaneously or because they have been convected by actin treadmilling to the minus-end of their actin track. Free myosins on the left-side of the two cluster system are more likely to bind to a red filament due the higher local prevalence of these filaments. Once bound to a red actin filament, these myosins are transported to the right by actin treadmilling. This cyclic flux implies a violation of detailed balance and underpins the active nature of the underlying processes. In fact, the steady-state analytical solution from eq. (S10) also describes the transient behavior in our simulations of acto-myosin bundles, see figure S5A.

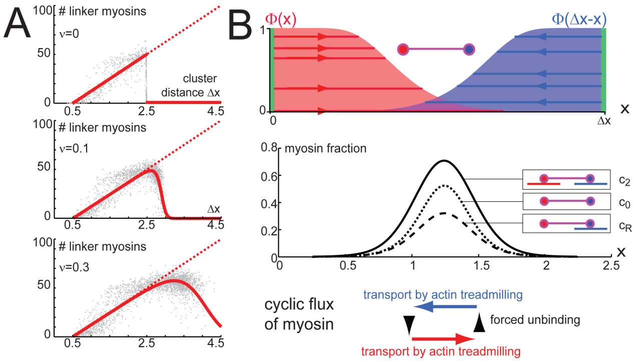


**Figure S5. Myosins crosslink actin clusters. A.** Myosin filaments mechanically link neighboring actin clusters by binding to one actin filament from each cluster, respectively. The plots show that in simulations of an acto-myosin bundle with variable actin filament length, the number of these linker myosins depends on the distance $\Delta x$ between actin clusters in a non-monotonic way (gray dots). Also shown is an analytical result for the number of linker myosin derived for a pair of static actin clusters at steady state (red), assuming that the total number of myosins enclosed by the two cluster centers scales as $c_m(\Delta x - L_m)$ where $c_m = N_m/L_{\mathrm{sys}}$ is the density of myosin filaments in the bundle (red dashed curve). Parameters as in figure 4 for different values of the length variability parameter, $\nu = 0,\ 0.1,\ 0.3$; simulation time, $t < 10$. For the mean field theory, we assume $N = 100$ actin filament per half-cluster. **B.** For the analytical theory, we consider an idealized scenario with two static actin half-clusters (with a certain length distribution of actin filaments) as well as a number of myosin filaments enclosed between the two cluster centers, see section S5. Myosin filaments can be either bound to one actin filament from each cluster (no motion due to force balance), be bound to an actin filament from one cluster only (myosin is convected by actin treadmilling), or be unbound. Using a mean field description, we can compute the fractions of myosins in the different binding states at steady state. This steady state is characterized by a cyclic flux of myosin filaments between the different binding states, see main text. Parameters: Actin filament number per half-cluster, $N = 5$ (for illustration purposes); cluster spacing, $\Delta x = 2.5$; actin length variability parameter, $\nu = 0.3$; myosin binding rates $k_{\mathrm{on}}$ and $k_{\mathrm{off}}$ as in table S1.
